# Familial Disclosure and Cascade Testing in High-Risk Families is Influenced by Gene Variant Penetrance: Implications for Family based Breast Cancer Prevention

**DOI:** 10.21203/rs.3.rs-9610726/v1

**Published:** 2026-05-05

**Authors:** Achille V.C. Manirakiza, Jincong Q. Freeman, Fangyuan Zhao, Emma Keel, Christine Drogan, Iris Romero, Dezheng Huo, Olufunmilayo I. Olopade

**Affiliations:** 1 Center for Innovation in Global Health & MaCLean Center for Clinical Medical Ethics, The University of Chicago, Chicago, IL; 2 Oncology Service, Department of Medicine, King Faisal Hospital Rwanda, Kigali, Rwanda; 3 Africa Health Sciences University, Kigali, Rwanda; 4 Department of Public Health Sciences, The University of Chicago, Chicago, IL; 5 Cancer Prevention and Control Research Program, The University of Chicago Medicine Comprehensive Cancer Center, Chicago, IL; 6 Center for Health and the Social Sciences, The University of Chicago, Chicago, IL; 7 Department of Obstetrics and Gynecology, University of Chicago Medicine, Chicago, IL; 8 Section of Hematology/Oncology, Department of Medicine and Center for Innovation in Global Health, The University of Chicago, Chicago, IL

## Abstract

**Background and Methods:**

Point of care genetic testing has grown but cascade testing of At-Risk Relatives (ARR) remains underutilized. Between July and September 2023, we surveyed 912 individuals enrolled in the Cancer Prone Study, a registry of diverse high-risk families who received genetic counseling from cancer genetic experts. We analyzed correlates of familial disclosure and cascade testing among carriers of pathogenic/likely pathogenic variants (PV). Data on carrier status of PV in cancer-predisposing genes, penetrance level of variants, receipt of a family letter, familial disclosure, and follow up cascade testing of ARRs were collected.

**Results:**

Of 912 respondents, 39.4% were carriers of PV. Among these carriers, 78% had highly penetrant PVs (most common genes were *BRCA1*: 39% and *BRCA2*: 18%) and 17.5% had moderate penetrance PVs; Rate of disclosure of PV to at least one family member; receipt of family letter explaining genetic test results; and sharing of information with ARRs were 98%, 69% and 82% respectively. Cascade testing was more likely among carriers with highly penetrance PV (91.1%) compared to those with moderate penetrance PV (58.3%) (adjusted odds ratio [AOR], 5.70; 95% CI, 2.03–16.02), and among carriers who received a family letter than among those who did not receive (87.2% vs. 73.0%; AOR, 3.70; 95% CI, 1.42–9.61). These data suggest that access to family-based counseling facilitated by cancer genetic experts improves understanding of familial risks and improves cascade testing following results disclosure. Our data support family based clinical interventions to optimize precision prevention and cancer control in high-risk individuals.

## Introduction

Factors driving familial disclosure and cascade genetic testing in cancer-prone families have been explored at length.^1–4^ Demographic features, including race/ethnicity, sex and age of the probands, along with the nature of pathogenic/likely pathogenic variants (PV/LPV) of cancer predisposition genes (CPGs), present in a given family are known to be influential and predictive of subsequent health-seeking behaviors.^1–5^ Probands – defined as the first individuals identified with a specific trait or disorder, serving as a starting point for a genetic study in a family – are now known to disclose their status to their at-risk relatives are reported rate of 30–97%.^4–6^ These disparities are largely defined by several factors, including demographic characteristics of the probands, their social backgrounds and prevailing familial dynamics.^5, 6^ For example, the choice of at-risk relatives to inform and the content of the discussion depend on the familial proximity (first- or second-degree relatives) and ease of communication within the families. Further, it is now understood that female probands are likely to disclose their genetic test results to their first-degree female relatives of the same generation.^1, 7^ As a consequence, cascade testing rates differ across healthcare settings and depend on sociodemographic factors, but also the perceived CPGs-conferred risk.^3, 8–12^

Well-studied CPGs, e.g., *BRCA1* or *BRCA2*, have historically prompted longer genetic counseling sessions and emphasis to probands, leading to increased rates of disclosure and cascade testing.^1,13,14^ This phenomenon is further demonstrated by the reported uncertainty associated with the presence of other, less known CPGs (e.g., *ATM* or *CHEK2*).^15–17^ Patients with PV/LPVs in moderate penetrance genes report being less worried about cancer risk, but more indecisive about sharing genetic test results with their family members, which is further compounded by the limited evidence regarding risk management recommendations from healthcare providers.^17^ Genetic cancer risk assessment programs offer both risk assessment and management for individuals at high risk for cancer. As these programs are multidisciplinary in nature, there is evidence of a missed opportunity to comprehensively raise awareness of cancer susceptibility through improved risk communication strategies.^6, 18^ In this study of high-risk individuals from a single academic center’s genetic cancer risk assessment program, we sought to examine 1) the various drivers and patterns of risk communication from probands to their at-risk relatives and 2) the relationship between receptor of a family letter and subsequent cascade testing among carriers of PV/LPV in CPGs.

## Methods

### Study Sample

Between July and September 2023, we conducted a survey among individuals aged ≥18 years enrolled in the University of Chicago Cancer Prone Study (CCPS). CCPS is a clinical registry initiated in 1992 that has enrolled 5,685 cancer high-risk participants as of September 2024. We sent 2,404 email invitations and 611 mail invitations to individuals with contact information in the registry. There were 325 undeliverable addresses. A total of 912 participants responded to the questionnaire. All study participants provided written informed consent to the CCPS and follow-up surveys. This survey study was reviewed and approved by the University of Chicago Institutional Review Board. We followed the Strengthening the Reporting of Observational Studies in Epidemiology (STROBE) reporting guidelines.

### Measures

The main independent variable was carrier status of PV/LPV in CPGs, which was assessed by asking participants whether they had been told by a genetic counselor or clinician that they had a gene variant(s) or change(s) increasing the risk of developing cancer. Genes were classified as either highly or moderately penetrant based on known clinical actionability and disease-specific risk (Supplementary Table 1).

In this study, probands, defined as PV/LPV carriers, were asked 1) the PV/LPV they carry; 2) whether they had disclosed the identified PV/LPV to any of their family members; and 3) whether they had received a family letter from their genetic counselor or clinician after genetic testing. The family letter was offered to probands to facilitate communication between the probands and their families, and contained information about the disease condition, the identified variant, and explanations of genetic testing. For participants who received a family letter, we also asked whether they had shared the letter with any of their family members.

We analyzed the patterns of genetic result disclosure through family letter sharing per family degree (did not disclose, disclosed to first-degree relatives, vs. disclosed to extended family) in relation to the proband according to standard guidelines. In brief, first-degree relatives were biological parents, full siblings and children of the probands. Extended family were defined as those who were related to the probands by two generational links (i.e., grandparents, aunts, uncles, and cousins). We also analyzed cascade testing, defined as genetic testing performed for any family member for the familial deleterious gene variant as a result of the familial disclosure. For this purpose, the genetic testing referral (clinical visits with a genetic counselor or testing directly from a laboratory) and direct consumer service (Ancestry, 23andme, etc.) were assessed. Additionally, carriers were asked whether their doctors or health care providers delivered the positive genetic test results during an in-person clinic visit or via telehealth.

Demographic and SES characteristics included age at survey, sex assigned at birth (female or male), race/ethnicity (non-Hispanic Asian, non-Hispanic Black, non-Hispanic White, and Hispanic), marital status (married or living with a partner, single or never married, divorced or separated, or widowed), highest level of education (high school or less, post-high school or some college, Associate’s degree, Bachelor’s degree, and graduate or professional degree), type of insurance (Medicaid, Medicare, private, other, or uninsured), and annual household income (<$50,000, $50,000 – $74,999, $75,000 – $99,999, $100,000 – $149,999, $15,000 – $199,999, or ≥$200,000) (See survey instrument).

### Statistical Analysis

Sample characteristics were described using summary statistics, overall, by CPGs carrier status, and by status of familial disclosure. Means (SD) and medians (IQR) were calculated for continuous variables, and counts and percentages were tabulated for categorical variables. Bivariate analyses were performed to compared exposure groups, using *t*-tests, Wilcoxon rank-sum tests, Chi-squared tests, ANOVA, or Fisher’s exact tests, as appropriate. Logistic regression was conducted to assess the association between receipt of a family letter and genetic testing status of any family members, controlling for level of penetrance gene, age at survey, sex assigned at birth, race/ethnicity, marital status, and highest level of education. Adjusted odds ratios (AOR) and 95% confidence intervals (95% CI) were computed. All statistical tests were two-sided, with a *P* value of <.05 considered statistically significant. Data were analyzed using Stata, version 18.0 (StataCorp LLC, College Station, TX) from November 2023 to May 2024.

## Results

### Sample Characteristics

Table 1 provides details on participants’ characteristics. There was a total of 912 survey respondents, with a mean age of 55.4 years (SD, 14.0). Overall, 39.4% (n=333) reported carrying a PV/LPV. 3.2% were non-Hispanic Asian, 10.3% were non-Hispanic Black, 82.7% were non-Hispanic White, and 3.9% were Hispanic. 44.3% had a graduate or professional degree; 30.7% reported having an annual household income of ≥$200,000; and 63.3% and 31.4% were privately insured and on Medicare/Medicaid, respectively.

Supplementary Figure 1 provides details of the PV/LPV distribution. Among individuals with a PV/LPV, 39.0% were in *BRCA1* (n=70 [21.0%]) or *BRCA2* (n=60 [18.0%]), followed by *CHEK2* (n=24 [7.2%]), *APC* (n=18 [5.4%]), *ATM* (n=18 [5.4%]), *MSH2* (n=15 [4.5%]), *PALB2* (n=13, [3.9%]), *MLH1* (n=12 [3.6%]), and *MSH6* (n=12 [3.6%]).

### Genetic Counseling, Familial Disclosure, and Cascade Testing

Most participants (74.9%) received their genetic testing information during an in-person visit with a genetic counselor. Participants who had their results through telehealth were younger than those who had in-person visits (mean [SD]: 50.7 [13.1] vs. 55.6 [13.9] years; *P*=.009). A higher proportion of Medicaid beneficiaries (37.5%) reported telehealth visits as compared to those privately insured (27.1%) or Medicare beneficiaries (16.7%) (*P*=.010) (Supplementary Table 2).

Among PV/LPV carriers, 68.7% reported having received a family letter from their genetic counselors to share with any family member; and of these, 82.2% reported having shared the family letter with at least one family member. No demographic or SES factors were significantly associated with family letter receipt (Supplementary Table 3).

Almost all (97.7%) PV/LPV carriers disclosed their results to at least one family member. Extent of disclosure was significantly associated with gene penetrance, with those carrying a PV/LPV in a high penetrance gene being much more likely to disclose to extended family members than those with a PV/LPV in a moderate penetrance gene (69.1% vs. 44.3%; *P*=.003) (Table 2).

Individuals who disclosed to extended family members were relatively younger (mean [SD]: 52.9 [13.4] vs. 57.1 [14.1] years; *P*=.02) than those who only disclosed to spouse or first-degree relatives. Furthermore, 83.5% of the individuals with a PV/LPV reported that their family members underwent genetic testing, with no significant differences across racial/ethnic groups. Having received a family letter during genetic counseling (87.2% vs. 73.0%; AOR, 3.70; 95% CI, 1.42–9.61) or carrying a high penetrant gene (vs. moderate penetrant gene: 91.1% vs. 58.3%; AOR, 5.70; 95% CI, 2.03–16.02) was associated with cascade genetic testing (Table 3).

## Discussion

Our study findings suggest several themes that are important in the context of evolving genetic cancer risk testing and counseling. We observed a high rate of familial disclosure in our patient population, which was strongly associated with the use of a family letter to aid this process. We observed that the level of penetrance of familial CPG is key in the uptake to cascade genetic testing by the probands’ relatives.

Access to cancer genetic services has been largely linked with sociodemographic factors. Previous studies demonstrated that patients with a racial/ethnic minority background and no insurance tend not to be referred to genetic counseling and testing.^22–26^ Several initiatives have been proposed with varying levels of success, such as primary provider education, point of care testing, and culturally tailored genetic counseling communication to increase minority participation in genetic counseling and testing. Leveraging telehealth could further reduce age disparities in access to genetic counseling as shown in our study, which correlates with literature regarding this mode of risk communication and traditional in-office genetic counseling for cancer risk. This suggests a similarity between in-person and telehealth visits, as well as the age influence on either choice.^28–33^ Additional tailored methods need to be investigated to increase the reach of cancer genetic services to various sociodemographic segments. For instance, initiating discussions in collective fora, with a strong technology emphasis, before progressing to individual consultations has demonstrated effectiveness in reducing anxiety and improving the uptake, disclosure of genetic risk, and cascade testing.^34–35^

Genetic information disclosure is generally high among family members and the prevalence of cascade genetic testing is high in our study population, consistent with previous research.^2–4^ To increase the uptake on familial disclosure, family letters are part of interventions that provide enhanced and structured gene-specific information and an opportunity for direct contact with healthcare providers. This method has resulted in high acceptability familial rates, and is now considered as a standard of care.^36–38^ The differences in cascade testing based on gene penetrance showcase the importance given well-known genes starting from genetic counseling sessions to family communication and disclosure content. We found that carriers with PV/LPV in high penetrance genes were associated with more disclosure to extended family members and increased cascade testing. This finding is consistent with Makhnoon et al. who reported longer and detailed genetic counseling content to *BRCA1* or *BRCA2* gene variants compared to other less known genes.^39^ The motivation to test for lesser studied genes has been explored in probands’ relatives found with breast cancer moderate risk genes, which was found to be driven by the need to understand personal risk.^15–17^ Uncertainty of management deters engagement in cancer risk assessment programs.^15–17^ Genetic cancer risk assessment is appropriate and concise in communicating risk among individuals with CPGs and enhances familial disclosure and cascade genetic testing.

Integrating Polygenic Risk Scores (PRS) in cancer risk assessment could provide an alternative for risk refinement for families with moderate penetrance genes and unaffected relatives. Data from the WISDOM trial demonstrated that risk-based screening approach – using PRS, genetics and lifestyle factors – is non-inferior to standard annual screening in high-risk individuals, while it reduces over-screening for low-risk individuals^40^. However, the clinical utility of PRS depends heavily on patient comprehension and equity. In a multi-ethnic cohort, high interest in the technology was coupled with a low overall awareness^41^. Addressing these literacy and equity barriers through structured familial communication is essential to solve the clinical ambiguity of moderate penetrance genes, and to ensure that the benefits of precision prevention are shared across all populations.

### Limitations

This study has several limitations. First, our results may not be representative of all high-risk individuals, based on the 40% response rate and the clinic setting (urban medical center). However, the prevalence estimates are congruent with existing literature. Second, data were per patient self-report, which is subject to recall bias or social desirability bias. However, we would expect this bias to be very minimal because our research staff had limited-to-no direct interaction with study participants at the time of the survey. This was unlikely to have influenced their survey responses. Moreover, self-reported PV/LPVs were verified independently by the investigators to ensure accuracy. Third, other unmeasured factors, e.g., perceived cancer risk, cultural belief or family background, and health-seeking behavior, may contribute to the findings observed in the study. It is worth exploring whether these factors impact genetic testing, familiar disclosure, cancer screening, and risk-reduction strategies in future research.

## Conclusions

In this diverse cohort of individuals at high risk for cancer, familial disclosure of CPGs carrier status and cascade testing were high. The penetrance level of genes and receipt of a family letter were associated with disclosure to extended family and genetic testing among carriers’ family members. Understanding familial disclosure and cascade testing from a gene-to-gene basis, particularly considering the motivation of the participants with high penetrance genes, could help improve genetic testing and counseling and risk communication among high-risk individuals. Our findings strongly suggests that a medical home is essential for family-based intervention studies in high-risk patients. This model promotes high adherence and follow-up, attributable to a structured clinical follow-up to coordinate and manage compliance.

Future studies are needed to evaluate how penetrance level of genes, familial disclosure and testing influence population cancer screening and prevention services for high-risk individuals.

## Supplementary Material

Supplement 1

## Figures and Tables

**Table 1.** Characteristics of Individuals at High Risk for Cancer, Overall and by Carrier Status of Cancer-predisposing Genes

		Carrier status of cancer-predisposing genes		
Characteristic	Overall (N = 912)	Non-Carriers (n = 513 [60.6%])	Carriers (n = 333 [39.4%])	
	N (%)	n (%)	n (%)	*P* value ^a^
**Age in years**				
Mean (SD)	58.2 (13.5)	60.0 (13.0)	55.4 (14.0)	<.001
Median (IQR)	60.0 (48.0–68.0)	61.0 (51.0–70.0)	57.0 (44.0–65.0)	<.001
**Sex assigned at birth**				
Male	179 (19.6)	92 (17.9)	73 (21.9)	.15
Female	733 (80.4)	421 (82.1)	260 (78.1)	
**Race/ethnicity**				
Non-Hispanic Asian	28 (3.2)	15 (3.0)	11 (3.4)	.03
Non-Hispanic Black	91 (10.3)	58 (11.7)	18 (5.5)	
Non-Hispanic White	735 (82.7)	405 (81.5)	285 (86.9)	
Hispanic	35 (3.9)	19 (3.8)	14 (4.3)	
**Marital status**				
Married or living with a partner	647 (72.8)	353 (71.2)	251 (76.8)	.13
Single or never married	93 (10.5)	50 (10.1)	32 (9.8)	
Divorced or separated	105 (11.8)	62 (12.5)	34 (10.4)	
Widowed	44 (5.0)	31 (6.3)	10 (3.1)	
**Highest level of education**				
High school/GED or less	65 (7.3)	35 (7.0)	25 (7.6)	.95
Post-high school or some college	103 (11.6)	51 (10.2)	38 (11.6)	
Associate’s degree	56 (6.3)	29 (5.8)	20 (6.1)	
Bachelor’s degree	273 (30.7)	157 (31.5)	97 (29.6)	
Graduate or professional degree	395 (44.3)	226 (45.4)	148 (45.1)	
**Type of health insurance**				
Private	562 (63.3)	300 (60.4)	225 (69.2)	.001
Medicaid	16 (1.8)	3 (0.6)	10 (3.1)	
Medicare	263 (29.6)	169 (34.0)	74 (22.8)	
Other/Other government plan	37 (4.2)	20 (4.0)	13 (4.0)	
Uninsured	10 (1.1)	5 (1.0)	3 (0.9)	
**Annual household income**				
<$50,000	92 (11.0)	45 (9.7)	35 (11.2)	.57
$50,000 – $74,999	93 (11.1)	51 (11.0)	34 (10.9)	
$75,000 – $99,999	110 (13.1)	55 (11.8)	45 (14.4)	
$100,000 – $149,999	168 (20.1)	103 (22.2)	55 (17.6)	
$150,000 – $199,999	118 (14.1)	64 (13.8)	49 (15.7)	
≥$200,000	257 (30.7)	147 (31.6)	95 (30.4)	
**Level of penetrance gene**				
Moderate	65 (21.9)	NA	65 (21.9)	NA
High	232 (78.1)	NA	232 (78.1)	

Abbreviations: SD, standard deviation: IQR, interquartile range; GED, general education development; NA, not applicable.

a *P* values were calculated using Student’s t, Wilcoxon rank-sum, Chi-squared, or Fisher’s exact tests, as appropriate.

**Table 2.** Distributions of Familial Disclosure of Gene Variant Status among Carriers, by Sociodemographic Factors

	Status of Disclosure			
Characteristic	Did not disclose	Disclosed to spouse and first-degree relatives	Disclosed to extended family	
	n (row %)	n (row %)	n (row %)	*P* value ^a^
**Age in years**				
Mean (SD)	59.7 (10.7)	57.1 (14.1)	52.9 (13.4)	.02
Median (IQR)	60 (54.0–69.0)	57 (47.0–68.0)	54 (42.0–64.0)	.02
**Sex assigned at birth**				
Male	0 (0.0)	29 (44.6)	36 (55.4)	.09
Female	7 (2.8)	78 (32.1)	158 (65.1)	
**Race/ethnicity**				
Non-Hispanic Asian	1 (9.1)	3 (27.3)	7 (63.6)	.08
Non-Hispanic Black	2 (14.2)	6 (42.9)	6 (42.9)	
Non-Hispanic White	4 (1.5)	92 (34.6)	170 (63.9)	
Hispanic	0 (0.0)	4 (30.8)	9 (69.2)	
**Marital status**				
Married/Living with partner	4 (1.7)	81 (34.0)	153 (64.3)	.32
Single/Never married	1 (3.4)	10 (34.5)	18 (62.1)	
Divorced/Separated	2 (6.4)	10 (32.3)	19 (61.3)	
Widowed	0 (0.0)	5 (62.5)	3 (37.5)	
**Highest level of education**				
High School/GED or less	0 (0.0)	6 (33.3)	12 (66.7)	.20
Post High School or Some College	1 (2.7)	7 (18.9)	29 (78.4)	
Associate’s degree	1 (5.3)	4 (21.0)	14 (73.7)	
Bachelor’s degree	3 (3.2)	38 (40.9)	52 (55.9)	
Graduate or Professional degree	2 (1.4)	52 (36.9)	87 (61.7)	
**Type of health insurance**				
Private	5 (2.3)	74 (33.9)	139 (63.8)	.29
Medicaid	0 (0.0)	0 (0.0)	8 (100.0)	
Medicare	1 (1.6)	27 (42.2)	36 (56.2)	
Other government plan	0 (0.0)	5 (41.7)	7 (58.3)	
Uninsured	0 (0.0)	0 (0.0)	3 (100.0)	
**Annual household income**				
<$50,000	0 (0.0)	10 (34.5)	19 (65.5)	.31
$50,000 – $74,999	1 (3.2)	8 (25.8)	22 (71.0)	
$75,000 – $99,999	2 (4.6)	14 (32.6)	27 (62.8)	
$100,000 – $149,999	1 (2.0)	14 (27.4)	36 (70.6)	
$150,000 – $199,999	1 (2.1)	13 (27.7)	33 (70.2)	
≥$200,000	2 (2.2)	43 (46.2)	48 (51.6)	
**Level of penetrance gene**				
Moderate	1 (1.9)	28 (53.8)	23 (44.3)	.003
High	2 (0.9)	66 (30.0)	152 (69.1)	

Abbreviations: SD, standard deviation: IQR, interquartile range; GED, general education development.

a *P* values were calculated using Student’s t, ANOVA, Kruskal-Wallis, Wilcoxon rank-sum, or Fisher’s exact tests, as appropriate.

**Table 3.** Family Letter Receipt and Level of Penetrance Gene Associated with Cascade Testing among Carriers’ Family Members

	Any family members had genetic testing for the gene variant(s) identified in you			Multivariable logistic regression
Characteristic	No (n = 47 [16.5%])	Yes (n = 238 [83.5%])		
	n (row %)	n (row %)	*P* value ^a^	AOR (95% CI) ^b^
**Received a family letter from your genetic counselor or other clinicians after your genetic testing**				
No	17 (27.0)	46 (73.0)	.01	1 [reference]
Yes	19 (12.8)	129 (87.2)		3.70 (1.42–9.61)
**Level of penetrance gene**				
Moderate	20 (41.7)	28 (58.3)	.001	1 [reference]
High	19 (8.9)	194 (91.1)		5.70 (2.03–16.02)
**Age in years**				
Mean (SD)	54.3 (12.3)	54.6 (14.2)	.89	1.30 (0.88–1.92) ^c^
Median (IQR)	53.0 (45.0–66.0)	56 (43.0–65.0)	.98	NA
**Sex assigned at birth**				
Male	12 (19.7)	49 (80.3)	.45	1 [reference]
Female	35 (15.6)	189 (84.4)		1.43 (0.46–4.38)
**Race/ethnicity**				
Non-Hispanic Asian	1 (11.1)	8 (88.9)	.81	2.98 (0.21–41.71)
Non-Hispanic Black	2 (18.2)	9 (81.8)		0.81 (0.08–8.27)
Non-Hispanic White	39 (15.7)	209 (84.3)		1 [reference]
Hispanic	3 (23.1)	10 (78.9)		0.15 (0.03–0.85)
**Highest level of education**				
High school/GED or less	4 (22.2)	14 (77.8)	.66	1 [reference]
Post-high school or some college	8 (22.2)	28 (77.8)		11.94 (0.88–161.47)
Associate’s degree	2 (11.1)	16 (88.9)		4.53 (0.49–42.23)
Bachelor’s degree	11 (13.3)	72 (86.7)		4.67 (0.83–26.43)
Graduate or professional degree	22 (16.9)	108 (83.1)		2.17 (0.49–9.54)
**Marital status**				
Married or living with a partner	36 (16.3)	185 (83.7)	.64	1 [reference]
Single or never married	3 (11.5)	23 (88.5)		0.72 (0.15–3.45)
Divorced or separated	6 (21.4)	22 (78.6)		0.66 90.14–3.16)
Widowed	2 (25.0)	6 (75.0)		0.22 (0.02–2.32)
**Type of health insurance**				
Private	34 (16.9)	167 (83.1)	.71	
Medicaid	0 (0.0)	8 (100.0)		
Medicare	10 (17.0)	49 (83.0)		
Other government plan	3 (25.0)	9 (75.0)		
Uninsured	0 (0.0)	3 (100.0)		
**Annual household income**				
<$50,000	2 (7.1)	26 (92.9)	.13	
$50,000 – $74,999	8 (28.6)	20 (71.4)		
$75,000 – $99,999	7 (18.4)	31 (81.6)		
$100,000 – $149,999	10 (20.4)	39 (79.6)		
$15,000 – $199,999	3 (7.0)	40 (93.0)		
≥$200,000	16 (18.6)	70 (81.4)		
**Did you share the family letter with any of your family members?** (among those who received a family letter)				
No	4 (22.2)	14 (77.8)	.27	
Yes	15 (12.3)	107 (87.7)		
**How did the family(s) receive the genetic testing?**				
Clinical visits with a doctor or generic counselor	NA	173 (78.7)	NA	
Used a consumer service (e.g., Ancestry, 23andme, etc.)	NA	8 (3.6)		
Received the test directly from a laboratory	NA	19 (8.6)		
Don’t know	NA	20 (9.1)		

Abbreviations: SD, standard deviation: IQR, interquartile range; GED, general education development; NA, not applicable.

a *P* values were calculated using Student’s t, Wilcoxon rank-sum, Chi-squared, or Fisher’s exact tests, as appropriate.

b Adjusted for receipt of a family letter, level of penetrance gene, age, sex assigned at birth, race/ethnicity, marital status, and highest level of education.

c The AOR (95% CI) for age was per 10-year increase.

## Data Availability

The current study used Individual-level patient data which cannot be shared publicly due to confidentiality and privacy concerns. However, the data can be de-identified and requested pending the approval of the University of Chicago Institutional Review Board and the corresponding authors.
