# Supplementary material for: Familial Disclosure and Cascade Testing in High-Risk Families is Influenced by Gene Variant Penetrance: Implications for Family based Breast Cancer Prevention
Source: Res Sq. 2026 May 5:rs.3.rs-9610726. Preprint. [Version 1] doi: 10.21203/rs.3.rs-9610726/v1 (PMC13174783; doi:10.21203/rs.3.rs-9610726/v1)
Supplement: Supplement 1 [file NIHPPrs9610726v1-supplement-1.pdf]

**Highly Penetrant Genes**	*BRCA1*, *BRCA2*, *APC*, *MLH1*, *MSH2*, *MSH6*, *PMS2*, *EPCAM*, *PALB2*, *PTEN*, *TP53*, *VHL*, *CDH1*, *CDKN2A*, *ALK*, *SMAD4*, *FLCN*, *MEN1*, *FH*, *MET*, *RUNX1*
**Moderately Penetrant Genes**	*ATM*, *CHEK2*, *APC** (p.I1307K), *SDHB*, *BARD1*, *MUTYH** (heterozygous), *RAD51D*, *BRIP*, *SDHC*, *DDX4*, *NBN*, *PRSS1*, *PDGFRA*, *MRE11A*, *WT1*, *HOXB13*

Supplementary Table 1. Categories of High and Moderate Penetrance Genes

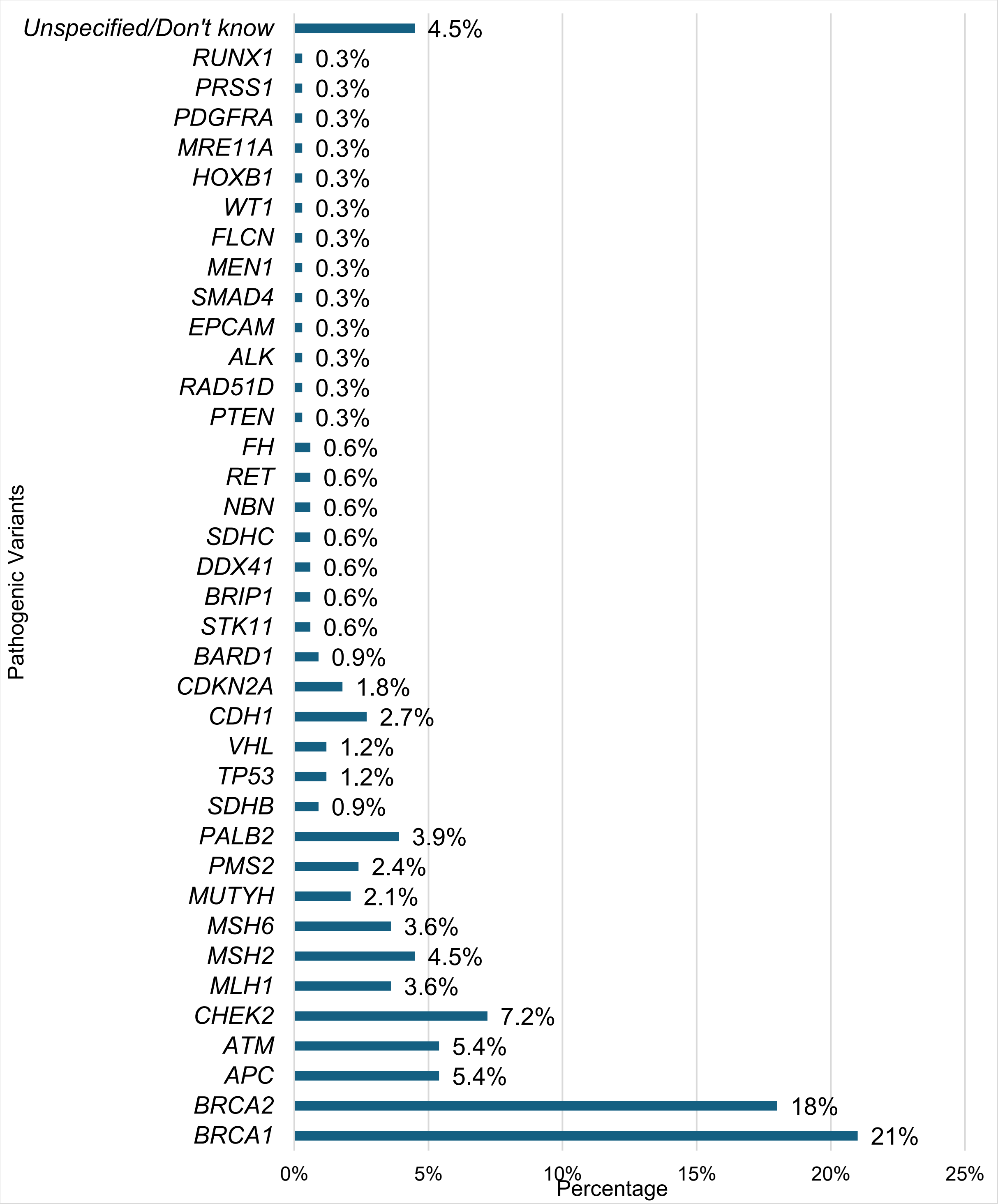

Unspecified/Don't know 4.5%
RUNX1 0.3%
PRSS1 0.3%
PDGFRA 0.3%
MRE11A 0.3%
HOXB1 0.3%
WT1 0.3%
FLCN 0.3%
MEN1 0.3%
SMAD4 0.3%
EPCAM 0.3%
ALK 0.3%
RAD51D 0.3%
PTEN 0.3%
FH 0.6%
RET 0.6%
NBN 0.6%
SDHC 0.6%
DDX41 0.6%
BRIP1 0.6%
STK11 0.6%
BARD1 0.9%
CDKN2A 1.8%
CDH1 2.7%
VHL 1.2%
TP53 1.2%
SDHB 0.9%
PALB2 3.9%
PMS2 2.4%
MUTYH 2.1%
MSH6 3.6%
MSH2 4.5%
MLH1 3.6%
CHEK2 7.2%
ATM 5.4%
APC 5.4%
BRCA2 18%
BRCA1 21%
Pathogenic Variants
0%
5%
10%
15%
20%
25%
Percentage

Supplementary Figure 1. Distributions of Reported Pathogenic Variants among Individuals Who Are Carriers

	Did your doctor or health care provider tell you the positive genetic test result via		
**Variable**	**In-Person visit (n = 221 [74.9%])**	**Telehealth visit (n = 74 [25.1%])**	
	**n (row %)**	**n (row %)**	***P* value [a]**
**Age in years**			
Mean (SD)	55.6 (13.9)	50.7 (13.1)	0.009
Median (IQR)	57.0 (45.0-65.0)	50.0 (40.0-62.0)	0.007
**Sex assigned at birth**			
Male	46 (78.0)	13 (22.0)	0.546
Female	175 (74.2)	61 (25.9)	
**Race/ethnicity**			
Non-Hispanic White	190 (74.8)	64 (25.2)	0.197
Non-Hispanic Black	12 (85.7)	2 (14.3)	
Non-Hispanic Asian	5 (50.0)	5 (50.0)	
Hispanic	12 (85.7)	2 (14.3)	
**Marital status**			
Married or living with a partner	164 (72.3)	63 (27.8)	0.074
Single or never married	22 (73.3)	8 (26.7)	
Divorced or separated	27 (90.0)	3 (10.0)	
Widowed	7 (100.0)	0 (0.0)	
**Highest level of education**			
High school/GED or less	17 (89.5)	2 (10.5)	0.609
Post-high school or some college	28 (77.8)	8 (22.2)	
Associate's degree	14 (77.8)	4 (22.2)	
Bachelor's degree	63 (74.1)	22 (25.9)	
Graduate or professional degree	99 (72.3)	38 (27.7)	

**Type of health insurance**			
Private	153 (72.9)	57 (27.1)	0.010
Medicaid	5 (62.5)	3 (37.5)	
Medicare	50 (83.3)	10 (16.7)	
Other/Other government plan	11 (91.7)	1 (8.3)	
Uninsured	0 (0.0)	3 (100.0)	
**Annual household income**			
<$50,000	22 (81.5)	5 (18.5)	0.495
$50,000 – $74,999	22 (81.5)	5 (18.5)	
$75,000 – $99,999	34 (81.0)	8 (19.1)	
$100,000 – $149,999	39 (76.5)	12 (23.5)	
$150,000 – $199,999	29 (64.4)	16 (35.6)	
≥$200,000	67 (74.4)	23 (25.6)	

Abbreviations: SD, standard deviation: IQR, interquartile range; GED, general education development.
[a] *P* values were calculated using Student's t, Wilcoxon rank-sum, Chi-squared, or Fisher's exact tests, as appropriate.

Supplementary Table 2. Distributions of Visit Type Regarding Genetic Test Result Disclosure among Carriers by Demographic Characteristics

	**Received a family letter from your genetic counselor or other clinicians after you genetic testing**		
**Variable**	**No (n = 72 [31.3%])**	**Yes (n = 158 [68.7%])**	
	**n (row %)**	**n (row %)**	***P* value [a]**
**Age in years**			
Mean (SD)	53.5 (12.3)	55.7 (13.2)	0.245
Median (IQR)	53.0 (44.0-64.0)	57 (45.0-65.0)	0.254
**Sex assigned at birth**			
Male	15 (31.3)	33 (68.7)	0.993
Female	57 (31.3)	125 (68.7)	
**Race/ethnicity**			
Non-Hispanic White	62 (30.8)	139 (69.2)	0.498
Non-Hispanic Black	2 (25.0)	6 (75.0)	
Non-Hispanic Asian	4 (57.1)	3 (42.9)	
Hispanic	3 (27.3)	8 (72.7)	
**Marital status**			
Married or living with a partner	59 (31.9)	126 (68.1)	0.439

Single or never married	7 (33.3)	14 (66.7)	
Divorced or separated	6 (35.3)	11 (64.7)	
Widowed	0 (0.0)	6 (100.0)	
**Highest level of education**			
High school/GED or less	2 (13.3)	13 (86.7)	0.381
Post-high school or some college	6 (26.1)	17 (73.9)	
Associate's degree	7 (46.7)	8 (53.3)	
Bachelor's degree	23 (32.9)	47 (67.1)	
Graduate or professional degree	34 (31.8)	73 (68.2)	
**Type of health insurance**			
Private	55 (34.0)	107 (66.0)	0.447
Medicaid	3 (50.0)	3 (50.0)	
Medicare	11 (22.9)	37 (77.1)	
Other government plan	2 (22.2)	7 (77.8)	
Uninsured	1 (33.3)	2 (66.7)	
**Annual household income**			
<$50,000	6 (27.3)	16 (72.7)	0.188
$50,000 – $74,999	8 (38.1)	13 (61.9)	
$75,000 – $99,999	8 (24.2)	25 (75.8)	
$100,000 – $149,999	10 (24.4)	31 (75.6)	
$15,000 – $199,999	8 (21.6)	29 (78.4)	
≥$200,000	28 (42.4)	38 (57.6)	
**Has a doctor told you that you had a cancer, leukemia or malignant tumor?**			
No	38 (31.9)	81 (68.1)	0.831
Yes	34 (30.6)	77 (69.4)	
**Did you share the family letter with any of your family members?**			
Yes	——	129 (82.2)	N/A
No	——	21 (13.4)	
Don't know	——	7 (4.5)	
**Degree relatives the family letter to whom shared with**			
First degree	——	75 (62.5)	N/A
Second degree	——	45 (37.5)	
**Level of penetrance gene**			
Moderate	12 (29.3)	29 (70.7)	0.766
High	51 (31.7)	110 (68.3)	

Abbreviations: SD, standard deviation: IQR, interquartile range; GED, general education development; N/A, not applicable.

[a] P values were calculated using Student's t, Wilcoxon rank-sum, or Fisher's exact tests, as appropriate.

Supplementary Table 3. Distributions of Family Letter Receipt among Carriers by Demographic Characteristic
